# Design of a Frequency Selective Rasorber Based on a Band-Patterned Octagonal Ring

**DOI:** 10.3390/ma16051960

**Published:** 2023-02-27

**Authors:** Xiaojun Huang, Yutao Ma, Xiaoyan Li, Linyan Guo, Helin Yang

**Affiliations:** 1 College of Communication and Information Engineering, Xi’an University of Science and Technology, Xi’an 710054, China; 2 College of Physical Science and Technology, Northwestern Polytechnical University, Xi’an 710129, China; 3 College of Geophysics and Information Technology, China University of Geosciences, Beijing 430074, China; 4 College of Physical Science and Technology, Central China Normal University, Wuhan 430079, China

**Keywords:** frequency selective rasorber, dual-polarization, low-profile, in-band, band-patterned octagonal ring

## Abstract

In this study, a dual-polarization and low-profile frequency-selective rasorber (FSR) constructed from a novel band-patterned octagonal ring and dipole slot-type elements is investigated. We show the process of designing from a full octagonal ring to realize a lossy frequency selective surface of our proposed FSR, and it has a passband with low insertion loss between the two absorptive bands. An equivalent circuit for our designed FSR is modeled to explain the introduction of the parallel resonance. Surface current, electric energy, and magnetic energy of the FSR are further investigated to illustrate the working mechanism. Simulated results indicate that *S*_11_ < −10 dB bandwidth within 5.2–14.8 GHz, *S*_21_ > −3 dB passband within 9.62–11.72 GHz, lower absorptive bandwidth within 5.02–8.80 GHz, and upper absorptive bandwidth within 12.94–14.89 GHz are obtained under normal incidence. Meanwhile, our proposed FSR possesses the properties of dual-polarization and angular stability. To verify the simulated results, a sample with thickness of 0.097 *λ*_L_ is manufactured, and the results are experimentally verified.

## 1. Introduction

Metamaterials have been the subject of intensive research in recent years [1,2,3,4]. Frequency selective surface (FSSs) can be described as a special case of a subgroup of planar metamaterials called metasurfaces. FSSs are used for spectral tailoring of the transmission, reflection, and absorption of electromagnetic (EM) waves. Thus, in essence, they perform filtering functions [5]. As a kind of spectral filter, FSSs are a kind of periodic two-dimensional pattern. They can be utilized to transmit EM waves in the operating bandwidth and reflect out-of-band EM waves in a different direction in a monostatic radar cross section (RCS) [6]. However, as radar detection techniques have advanced, this approach is no longer applicable because a multi-static radar system could detect the reflected EM waves easily. Thus, there is an idea that FSS can transmit passband EM waves while absorbing out-of-passband EM waves to improve the stealth performance [7]. In order to satisfy such an idea, frequency selective rasorber (FSR), as a kind of novel FSS structure, was conceived [8].

An FSR can be described as combination of a lossy layer containing lossy FSS and a bandpass layer containing lossless FSS, where the lossy FSS is generally realized by resistor-loaded metal surface elements [9,10,11,12,13,14,15,16,17,18,19,20], and the lossless FSS can be designed by metal slot-type elements [11,21,22] or strip elements [23,24]. An FSR typically needs to include a passband with an insertion loss (IL) of less than 3dB, and according to the relative positional relationship between the absorptive band and the passband, FSR can be classified as T-A, A-T, and A-T-A FSR, where T-A indicates that the passband is on the left side of the absorption band, A-T indicates that the passband is on the right side of the absorption band, and A-T-A indicates that the passband is between two absorption bands. Among them, the A-T-A FSR has received the most attention because its frequency response is more suitable for practical applications.

The lossy FSS of A-T-A FSR in reported works is mainly achieved through the combination with different basic elements, and these basic elements include strip lines [12], interdigital resonator [15], spiral ring [20], cross dipole [24], circular rings [25], cross grids [26], etc. The lossy FSS of the first A-T-A FSR proposed by Shang et al. was implemented by a combination of a square ring and a cross [23], however too many lumped elements were embedded in the structure. Huang et al. used two metallic, square loops, cross strip, and a few lumped resistors to realize a hybrid resonator [11]. Parameswaran et al. proposed a notch resonator containing diagonal and meander line slots to design the lossy FSS [27]. Luo et al. used double-sided parallel-strip lines and a circular ring to realize a lossy FSS [12]. Xing et al. used four rectangular spiral resonators and resistor-loaded metal lines to realize a lossy FSS [20]. Guo et al. used dipole-like elements and varactor diodes to realize an FSR with a tunable passband [28]. Bakshi et al. designed a configurable FSR by embedding varactor diodes and PIN diodes into FSS [29].

Studies on the FSRs indicate that a resonator in the lossy FSS is crucial for achieving desired features, such as a good passband, low profile, and angular stability. Many resonators have been designed to become parallel resonators in the passband, so these various designs are identical in principle. It is beneficial to pursue the simplicity of the design, because a simpler lossy FSS with fewer resistors can reduce the difficulty of manufacturing. In reported studies, some researchers designed a simple lossy FSS but used too many resistors, while others used fewer resistors, but their lossy FSS layout was complicated [10,12,27]. Still, others created simpler lossy FSS with fewer resistors using combined elements [11,20]. However, designing simpler lossy FSS still requires effort. Many existing works have utilized expensive substrate materials [12,25], which limit their use in some applications that require large areas of A-T-A FSR. Therefore, the selection of low-cost substrate materials can effectively reduce the cost of the application.

In this article, our goal is to create a lower-cost A-T-A FSR with a passband in the X-band. The design of the lossy FSS with a simple layout involves transforming a regular octagonal ring into a band-patterned octagonal ring. We provide the steps for designing and a clear explanation about the operation. Moreover, the thickness of our proposed FSR is low compared with other FSRs, and we use low-cost substrate FR4, which expands its application scenarios to some extent, and the proposed FSR can be used to improve the stealth performance of a radar system.

## 2. Design and Analysis

**Figure 1:**
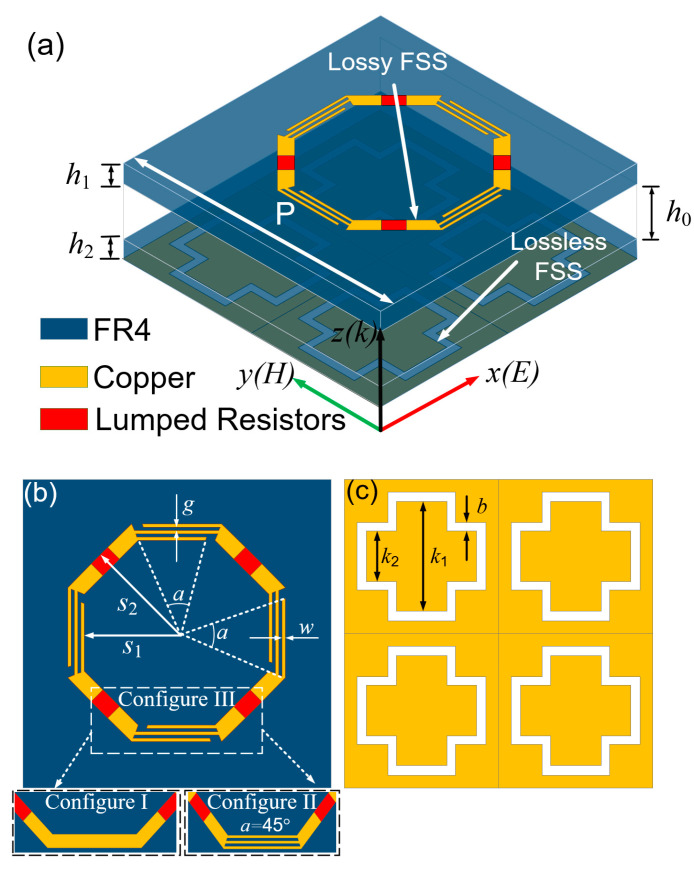
(**a**) Schematic of the FSR. (**b**) Lossy FSS. (**c**) Lossless FSS (geometrical dimensions: *P* = 14.6 mm, *h*_0_ = 3.6 mm, *h*_1_ = 1 mm, *h*_2_ = 1 mm, *s*_1_ = 3.98 mm, *s*_2_ = 4.68 mm, *w* = 0.14 mm, *g* = 0.14 mm, *α* = 40°, *k*_1_ = 5.2 mm, *k*_2_ = 2.4 mm, *b* = 0.4 mm; all lumped resistors are 120 Ω).

Figure 1a presents the unit cell structure of our FSR, and its overall view indicates that the unit cell consists of three layers. The top layer is a lossy layer including a lossy FSS printed on an FR4 dielectric substrate (ε_r_ = 4.3 and tanδ = 0.025), and the lossy FSS is a lumped resistor-loaded split octagonal ring made of copper. The bottom layer is a bandpass layer including a lossless FSS printed under an FR4 dielectric substrate as well, and the lossless FSS is a slot-type FSS made of a copper sheet. The middle layer is an air gap with a thickness of h_0_ sandwiched between the two dielectric substrates. The design concept of the lossy FSS involves transforming one of every two sides of the octagonal ring into three stripes along its length, while interrupting the connection between some strips with their adjacent sides. This concept is reflected well in Figure 1b, where *s*_1_ and *s*_2_ are the inner radius and the outer radius of the ring, respectively. The width of the strips is represented by *w* and the gap between every two strips is *g*. As a kind of regular convex polygon, the central angle of a regular octagon is 45°. It can be noticed that, for each of the three strips generated by the transformed side, the geometric parameters of the upper and lower strips are adjustable, except for the middle strip, which is fixed. The length of two adjustable strips and the distance of the two strips along their adjacent sides are controlled by angle *α* between the vertex of the strip and the geometric center of the octagonal ring. When α is equal to 45°, the stripes are connected to their adjacent sides; when α is less than 45°, a gap will be formed between the strip and its adjacent side. As *α* decreases, the distance of the gap will increase. Figure 1c presents the layout for the bandpass layer, and four dipole-type slots construct the lossless FSS in the unit cell.

**Figure 2:**
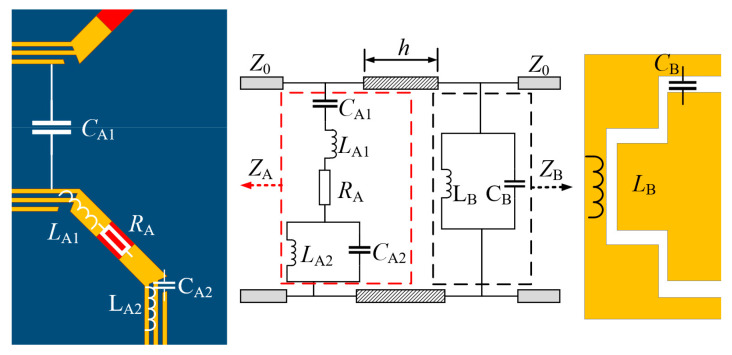
Equivalent circuit for the proposed FSR (optimized circuit parameters by ADS: *R*_A_ = 256 Ω, *C*_A1_ = 0.117 pF, *L*_A1_ = 1.834 nH, *C*_A2_ = 0.20 pF, *L*_A2_ = 1.183 nH, *L*_B_ = 1.022 nH, *C*_B_ = 0.23 pF, the electrical length *h* is 90° at 10.8 GHz).

The frequency response of our discussed FSR is A-T-A, and for an ideal design, there is no reflection (|*S*_11_| = 0) within the entire A-T-A band. At the same time, a unity transmission (|*S_2_*_1_*|* = 1) exists in the passband and no transmission (|*S*_21_| = 0) exists in the absorptive band. In general, this ideal condition is impossible to satisfy. Thus, in actual design and analysis, the reflection coefficient should be less than −10 dB (|*S*_11_| < −10 dB) and the transmission coefficient should be greater than −3 dB in the passband (|*S*_21_| > −3 dB). The proposed FSR can be equivalent to a two-part network, and its equivalent circuit is modeled in Figure 2. The circuit parameters are optimized by Advanced Design System (ADS). Lossless FSS with impedance *Z*_B_ can be represented by an LC parallel resonator (*L*_B_//*C*_B_). Within the absorptive band, |*S*_21_| needs to get close to 0, and the lossless FSS blocks the transmission of EM waves to perform as a ground plane; thus, the value of *Z*_B_ approaches 0. Meanwhile, the value of *Z*_B_ tends to infinity (*Z*_B_ → ∞) when the lossless FSS resonates at the passband because the parallel resonance exists in the slot-type lossless FSS. As analyzed in [15,21], when *Z*_B_ → ∞ within the passband, |*S*_21_| can be calculated as:

(1) $$\left|S_{21}\right|=\frac{2}{\left|2+Z_{0}/Z_{A}\right|}$$

where *Z*_0_ = 120π Ω represents the characteristic impedance of free space, and *Z*_A_ denotes the impedance of the lossy FSS. According to Expression (1), in order to obtain the value of |*S*_21_| close to 1 (|*S*_21_|→ 1), *Z*_A_ should approach infinity at the passband, so a strategy to realize parallel resonance in the lossy FSS can satisfy the goal of designing an FSR.

However, a common equivalent circuit of the lossy FSS is series RLC, which cannot satisfy the condition that |*S*_21_| → 1 in Expression (1) because no parallel resonance exists in the circuit. To overcome this limitation, a full regular octagonal ring is designed to the surface (Configure III), as shown in Figure 1b. The gap between the strip and its adjacent side can form an equivalent capacitance *C*_A2_ and the equivalent inductance of the strips is *L*_A2_. As a result, a parallel resonator (*L*_A2_//*C*_A2_) is introduced into the lossy FSS. *C*_A1_ represents the coupling capacitance between unit cells, *L*_A1_ is the equivalent capacitance of the ring, and *R*_A_ represents the entire equivalent impedance on the lossy FSS. Moreover, when the EM wave is incident on the FSR, three phenomena, reflection, transmission, and absorption, occur, where |*S*_11_|^2^ denotes the reflectivity and |*S*_21_|^2^ denotes the transmittance, and then the absorptivity of the FSR is defined as

(2) $$A\left(f\right)=1-{\left|S_{11}\right|}^{2}-{\left|S_{21}\right|}^{2}$$

## 3. Simulation Results and Discussion

**Figure 3:**
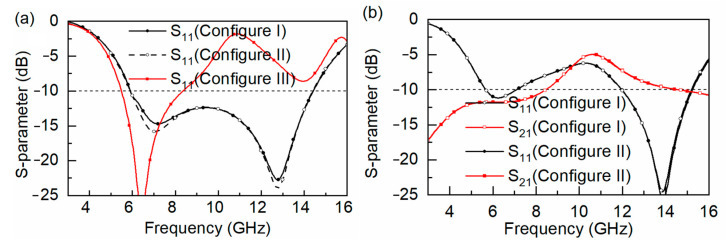
(**a**) *S*_11_ when lossless FSS is replaced by ground plane (**b**) *S*_11_ and *S*_21_ under Configuration I and II with lossless FSS.

In this section, numerical simulations are performed by using full-wave simulation CST software. Additionally, in CST, the solver we used is the frequency-domain solver. Unit cell boundary conditions are set in the *x*-*y* plane, and the Floquet port are set along the *z*-axis. The mesh type and size are set to tetrahedral and adaptive size, respectively. Additionally, the lossy FSS end is set to port 1 (the incident port of the EM wave) and the lossless FSS end is set to port 2 (the transmission port of the EM wave). The simulated results of the *S*-parameters are shown for when the metal ground plane replaces the lossless FSS in Figure 1c, and the *S*_11_ curves under different configurations of lossy FSS are presented in Figure 3a. Configuration I represents the lossy FSS presented in the lower left corner of Figure 1b, which is an full regular octagonal ring, and the *S*_11_ < −10 dB bandwidth is 5.9–14.4 GHz under normal incidence. Configuration II represents the lossy FSS, which is the FSS presented in the lower right corner of Figure 1b, and the *S*_11_ curve is the same as in Configuration I, which reflects that both the lossy FSSs share the same equivalent circuit. Configuration III is realized by setting the value of α smaller than 45°, and a gap is formed between the strip with its adjacent side. It is clear that the *S*_11_ of the absorber undergoes a significant change compared with the Configuration I and II, since the parallel resonator *L*_A2_*C*_A2_ is introduced into the lossy FSS.

The simulated results of *S*_11_ and *S*_21_ for the FSR, including the lossless FSS, are presented in Figure 3b. Under Configuration I and II of the lossy FSS, the return loss of FSR is below 10 dB and the IL of FSR is above 3 dB, which is far away from the necessary A-T-A response for a desired FSR. Meanwhile, the *S*_11_ and *S*_21_ curves are basically the same for both cases, which reflects that the modification of just transforming the side of an octagonal ring into three strips without gaps is unnecessary.

**Figure 4:**
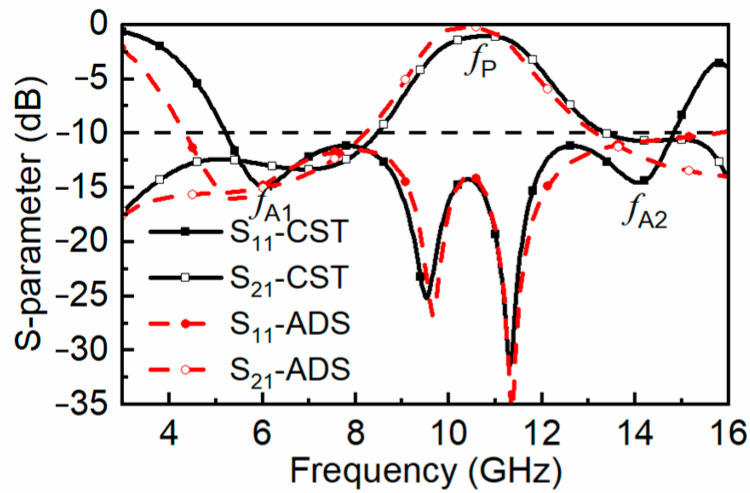
*S*-parameter and absorptivity of the proposed under normal incidence.

However, under Configuration III of lossy FSS, Figure 1b shows the layout of the lossy FSS in our proposed design. The *S*_11_ < −10 dB bandwidth is 5.2–14.8 GHz, and *S*_21_ is above −3 dB within 9.62–11.72 GHz; thus, the purpose of the modification of decreasing α is to generate a gap that is effective for the design of the FSR. Moreover, two absorption bandwidths within 5.02–8.80 GHz and 12.94–14.89 GHz are obtained. The dotted curves in Figure 4 show the fitted *S*-parameter curves of the equivalent circuit in Figure 2, which are basically consistent with the results of the full-wave simulation. The frequency point at 6 GHz within the lower absorptive band, the frequency point at 10.8 GHz within the transmission band, and the frequency point at 14 GHz within the upper absorptive band are marked as *f*_A1_, *f*_P_ and *f*_A2_, respectively.

**Figure 5:**
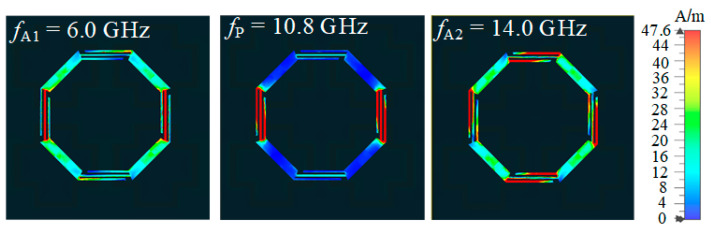
Surface current distributions of the lossy FSS at *f*_A1_, *f*_P_, and *f*_A2_.

**Figure 6:**
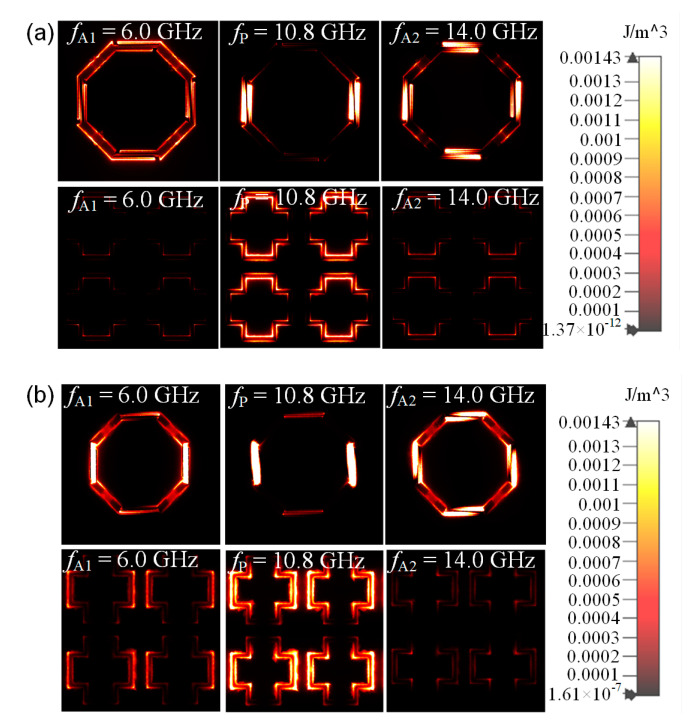
(**a**) Electric field energy and (**b**) magnetic field energy distribution under TE polarization of the lossy layer and bandpass layer at *f*_A1_, *f*_P_ and *f*_A2_.

Surface current distributions of the lossy FSS at *f*_A1_, *f*_P_, and *f*_A2_ are shown in Figure 5. It can be noticed that at the three frequency points, currents with the highest values appear on the strips. However, no surface current appears on the resistor-loaded sides in the passband, so ohmic loss generated by resistors does not occur, while at *f*_A1_ and *f*_A2_, surface currents flow through the resistors, so the absorption of EM waves occurs. As shown in Figure 6a, at *f*_A1_ and *f*_A2_, the electric field energy is weak on lossless FSS, while it is much stronger on the lossless FSS at *f*_P_. Similarly, as shown in Figure 6b, the distribution of magnetic field energy on the slot of the lossless FSS is significant in the passband. This observation indicates that the lossless FSS behaves as a ground plane in the absorptive band, so the lossless FSS blocks the transmission of EM waves, while EM waves can pass through in the passband. It is obvious that both the electric field energy and the magnetic energy field merely distribute on the strips at the frequency point *f*_P_ as no energy appears on the sides where resistors are embedded. At *f*_A1_ and *f*_A2_, the energy is concentrated on the sides in which the resistors are embedded, so the energy is absorbed.

**Figure 7:**
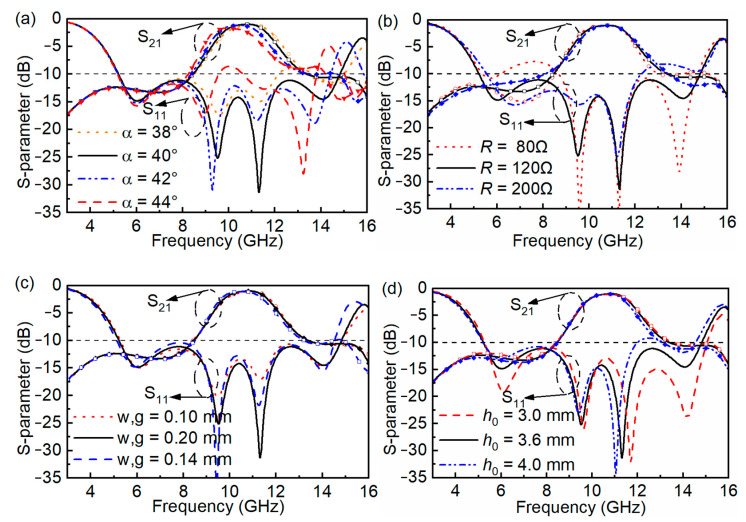
The result of *S*_11_ and *S*_21_ under different parameters. (**a**) Angle *α* of the band-patterned ring. (**b**) Lumped resistors R. (**c**) Width of strips *w* and gap *g* between each two strips. (**d**) Thickness *h*_0_ of air between the FR4 substrates.

The effects of different structural parameters on the proposed FSR are presented in Figure 7. Angle α is used to present the size of the gap between the strip and its adjacent side. The proposed FSR does not work when *α* = 45°. Figure 7a presents the *S*_11_ and *S*_21_ curves under different *α* values, and when *α* is 38° and 40°, the *S*_21_ curves are basically the same, while the *S*_11_ < −10 dB bandwidth is narrower when α is 38°. As α increases to 42° and 44°, the passband bandwidths (*S*_21_ > −3 dB) are narrowed. Thus, α = 40° is an optimal candidate to be selected. As presented in Figure 7b, the lumped resistors with different *R* values have less effect on *S*_21_ and 120 Ω, as an intermediate value brings a better *S*_11_ curve within 6–15 GHz. *S*_11_ fluctuates around −10 dB within part of the bandwidth when *R* is 80 Ω or 200 Ω. As presented in Figure 7c, there are no significant changes in *S*_11_ and *S*_21_ under the combinations of *w* and *g*. The air thickness of *h*_0_ has no influence on *S*_21_ because *S*_21_ is decided by the impedance of the lossy layer listed in Expression (1), and among the parameters shown in Figure 7d, only the parameter *h*_0_ has no influence on *Z*_A_.

**Figure 8:**
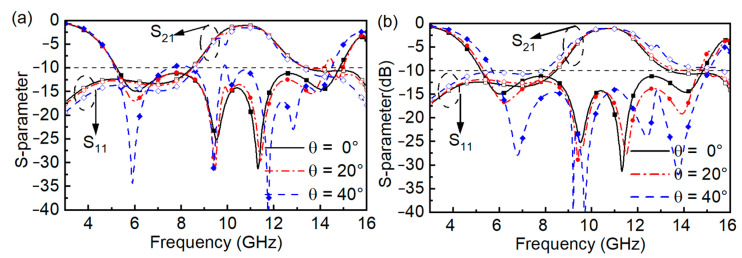
Simulated results of oblique incident. (**a**)TE polarization. (**b**)TM polarization.

Our proposed FSR has the superiority of dual polarization due to the property of rotational symmetric. As shown in Figure 8, under normal incidence, the *S*_11_ and *S*_21_ values of TE and TM polarizations are identical, where the bandwidth of *S*_11_ < −10 dB is 5.2–14.8 GHz, the bandwidth of *S*_21_ > −3 dB is 9.62–11.72 GHz, and at 10.8 GHz, a low IL of 1 dB is obtained. When the incident angle θ is equal to 20°, the bandwidth of *S*_21_ > −3 dB is 9.66–11.74 GHz under TE polarization, and the bandwidth of *S*_21_ > −3 dB is 9.61–11.82 GHz under TM polarization. When the incident angle θ is 40°, the bandwidths of *S*_21_ > −3 dB are 10.09–11.70 GHz and 9.52–12.04 GHz under TE polarization and TM polarization, respectively. Meanwhile, the change in bandwidths of *S*_11_ < −10 dB is small when the incident angle θ has different values of 0°, 20°, and 40° under both polarizations.

## 4. Experiment Verification

**Figure 9:**
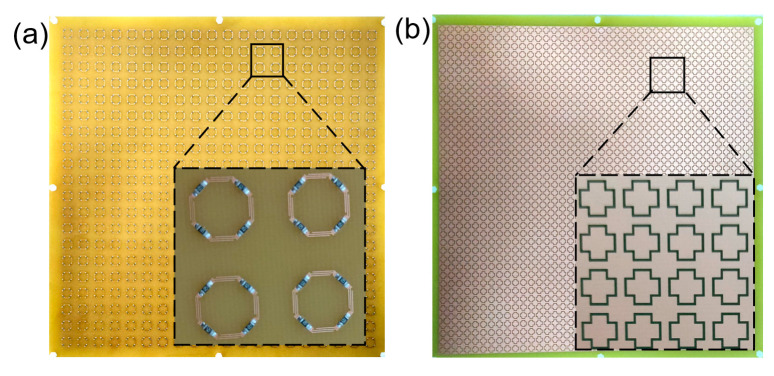

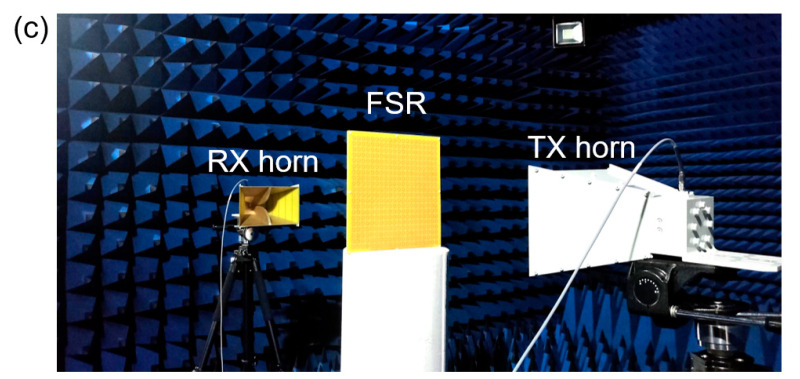
Fabricated sample and experimental system. (**a**) Lossy layer. (**b**) Bandpass layer. (**c**) Experimental system.

To verify the accuracy of the simulated aforementioned results, a sample of our proposed FSR is fabricated using the technology of a standard printed circuit board (PCB). The period of the sample is 14.6 mm, and the FR4 dielectric substrates with a thickness of 1 mm are used for both the lossy layer and bandpass layer. Here, we use 20 × 20 cells for experimental testing. Thus, 20 × 20 arrays are fabricated, and the sample is 312 × 312 × 5.6 mm^3^ in terms of overall dimensions. Figure 9a,b present a photograph of the lossy layer and bandpass layer, where the lossy layer and the bandpass layer are separated by nylon screws and nylon nuts to form a 3.6 mm thick air gap, and four chip resistors with a value of 120 Ω are welded on the lossy FSS in each unit cell. Additionally, in the bandpass layer, the slot-type periodic pattern is made by etching a complete metal plate. Figure 9c presents the experimental system in an anechoic chamber, where the main experimental instruments are a vector network analyzer (ROHDE&SCHWARZ) and two horn antennas. Furthermore, we can measure the *S*-parameter curves under polarizations of TE and TM at different incident angles of the fabricated sample by using the experimental system.

**Figure 10:**
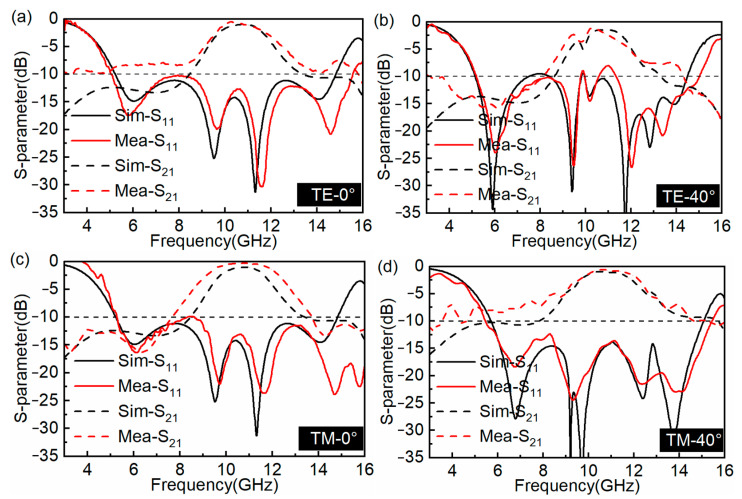
Measured and simulated results of the FSR. (**a**,**b**) TE polarization at θ = 0° and θ = 40° (**c**,**d**) TM polarization at θ = 0° and θ = 40°.

The comparison results of measured and simulated *S*_11_ and *S*_21_ curves are presented in Figure 10. We show the comparison results of experimental tests and simulations under TE and TM polarization at incident angles of 0° and 40°, respectively. It is obvious that the measured results have a good agreement with the simulated results. Therefore, this strongly validates the rationality of our proposed FSR. Moreover, Table 1 lists the comparisons between our proposed A-T-A FSR and the other FSRs. In the comparison, it can be found that the basic idea of designing lossy FSS in different research is to introduce parallel resonance, and by creating a band-patterned octagonal ring in the lossy layer, our proposed FSR possesses the excellent characteristics of low-profile and low IL at X-band.

## 5. Conclusions

In conclusion, we have designed a low-cost A-T-A frequency selective rasorber with a simple lossy FSS, and its passband was located at the X-band. During the design phase, we transformed a full regular octagonal ring to introduce parallel resonance in the lossy FSS. It can be proven that a regular octagonal ring or just transforming the side of the octagonal ring into strips for the lossy FSS cannot satisfy the desired frequency response of A-T-A FSR, and setting the angle α at less than 45° to form a gap was effective for the design since parallel resonance was introduced into the lossy FSS. A passband was obtained at 10.8 GHz and the *S*_21_ > −3 dB bandwidth was found at 9.62–11.72 GHz. The *S*_11_ < −10 dB spans from 5.2 to 14.8 GHz, and two absorption bandwidths 5.02–8.80 GHz and 12.94–14.89 GHz were obtained. Moreover, a sample with thickness of only 5.6 mm (0.097 *λ*_L_) was fabricated, and the measured results were well validated for the simulations, and the proposed FSR had characteristics of low IL, angular stability, dual-polarization, and low-cost.

## Figures and Tables

**Table 1:** Performance comparison of the previously reported FSR.

Ref	Realization of Lossy FSS	Passband(*f*_P_) ^a^	Absorption Band Upper/Lower ^b^	Angular Stability	Thickness (*λ*_L_)
[12]	Parallel-Strip Lines	1.24@6 GHz	2.63–5.19; 6.17–8.45	30°	9.8 mm/0.085
[15]	Interdigital Resonator	0.3@10.3 GHz	3.76–8.7; 12–16.08	45°	7 mm/0.24
[25]	Notch Structure	0.48@5.7 GHz	2.6–5.25; 6.05–8.5	35°	14 mm/0.15
[26]	LC resonators	2.45@6.74 GHz	3.61–6.12; 6.98–10.17	45°	9 mm/0.108
[30]	Thin copper wire	1.0@8.3 GHz	2.5–6.3; NA	45°	10 mm/0.090
[31]	Slot resonators	1.28@3.68 GHz	1.79–2.6; 4.5–5.25	40°	13 mm/0.153
Our work	Band-patterned octagonal ring	1.0@10.8 Gz	5.02–8.80; 12.94–14.89	40°	5.6 mm/0.097

^a^ Frequency point with minimum insertion loss. ^b^ Absorption bandwidth above 80% absorptivity.

## Data Availability

The data presented in this study are available on request form the corresponding author.
